# How Clinical Research Can Contribute to Strengthening Health Systems in Low Resource Countries

**DOI:** 10.3390/tropicalmed5020048

**Published:** 2020-03-29

**Authors:** Florent Mbo, Wilfried Mutombo, Digas Ngolo, Patrice Kabangu, Olaf Valverde Mordt, Nathalie Strub Wourgaft, Erick Mwamba

**Affiliations:** 1 HAT Platform, Drugs for Neglected Diseases initiative, Avenue Milambo, N 4, Quartier Socimat, Gombe, Kinshasa 7948, Democratic Republic of Congo; 2 Le Programme Nationale de Lutte contre la Trypanosomiase Humaine Africaine (PNLTHA), croisement du Boulevard Triomphal avec l’avenue de la Libération, Commune de Kasa-Vubu, Kinshasa 7948, Democratic Republic of Congo; dngolo@dndi.org (D.N.); pkabangu@dndi.org (P.K.); erickmwamb2002@yahoo.fr (E.M.); 3 Drugs for Neglected Diseases initiative (DNDi), Avenue Milambo, N 4, Quartier Socimat, Gombe, Kinshasa 7948, Democratic Republic of Congo; wmutombo@dndi.org; 4 Drugs for Neglected Diseases initiative, 15 Chemin Louis-Dunant, 1202 Geneva, Switzerland; ovalverde@dndi.org (O.V.M.); nstrub@dndi.org (N.S.W.)

**Keywords:** human African trypanosomiasis, clinical research, health system strengthening

## Abstract

Clinical research on neglected tropical diseases is a challenge in low-resource countries, and the contribution of clinical and operational research to health system strengthening is poorly documented. Developing new, simple, safe, and effective treatments may improve the effectiveness of health systems, and conducting research directly in health structures may have an additional impact. This study describes the process of conducting clinical trials in the Democratic Republic of Congo (DRC) in compliance with international standards, and the role of the trials in strengthening health system functions, including governance, human resources, health information, provision of care, and the equipping of health services with the necessary supplies and infrastructure. We conclude that conducting clinical trials in endemic areas has not only reinforced and supported the aim of conducting high-level clinical research in endemic countries, but has also brought lasting benefits to researchers, staff, and hospitals, as well as to broader health systems, which have positive knock-on effect on patients outside of the clinical trials and their communities. Sustainability, however, remains a challenge in an underfunded health system, especially with respect to specialized equipment. Clinical research in most of sub-Saharan Africa is highly dependent on international input and external technical support; there are areas of weaknesses in trial design and documentation, as well as in data management and analysis. Financing remains a critical issue, as African investigators have difficulties in directly accessing sources of international research funding.

## 1. Introduction

The scientific and social value of research can be difficult to quantify, but it is generally based on three factors:

- the quality of the information produced;
- its relevance to significant health problems;
- its contribution to the creation or evaluation of interventions, policies, or practices that promote individual or public health.

Research needs to show that it is responding to needs or priorities by demonstrating that new knowledge will be generated about the best ways to address a pathology present in the target community. It is possible to measure the impact of health system improvements brought to patients and their communities when clinical trials are conducted in low-resource settings [1].

The Council for International Organizations of Medical Sciences/World Health Organization International Ethical Guidelines for Health-related Research Involving Humans states that for health-related research involving humans to be ethically justified, there must be the prospect of generating knowledge and the means necessary to protect and promote people’s health. After successful completion of clinical research, the introduction of a new treatment into general practice may significantly increase the effectiveness of the health system itself. This is particularly true in the field of neglected tropical diseases, which is classified as “tool-deficient” and in need of well-adapted simpler, safer, and/or more effective treatments [2]. 

Human African trypanosomiasis (HAT) is a neglected tropical disease, predominating in remote areas of Africa where health services are sometimes deficient or poorly equipped. When communities or policymakers identify research on new treatment tools as a public health priority, plans for clinical trials to address this particular need should aim to bring social value to the affected population, and thus respond to broader health needs [1]. The degree to which this will be possible will depend on the relevance of the information that the study is intended to produce for the community in addition to the introduction of specialized training, equipment, and supplies. This approach adds value to the direct justification for the conduct of clinical trials for treatments for HAT. Managers of the national sleeping sickness control program and their research partners in endemic countries determined the target product profile for a new drug for HAT treatment in response to information gathered through interviews with physicians, technicians on the ground, and mobile teams who had experienced serious adverse reactions associated with the use of Melasoprol, such as encephalopathy. The review process, starting from a draft proposed by Drugs for Neglected Diseases initiative (DND*i*), involved two rounds of discussion in a general scientific meeting, followed by a restricted meeting in which each proposed product quality was debated and agreed on by the participants.

Benefits other than those directly associated with participation in the study may be devolved to communities or populations, particularly in low-resource settings. These benefits include improved health infrastructure, trained laboratory personnel, and education of the public about the nature of the research and the benefits of a study. Clinical trials on treatments for HAT also contribute to improvements in management by virtue of staff training and equipping the hospitals involved. [1] 

Sleeping sickness was historically managed separately within a strong vertical system, which was separated from the often-underfunded and ill-equipped general health service. The focus of the clinical trials is to address the primary need for developing simplified tools. Integrating NTD care into the general health system is a long process that risks a loss of quality unless the structures and staff are reinforced and supported. As clinical trials are limited in time, this process needs to concentrate on improving the capacity of health staff and providing adequate means for them to perform their roles. 

The objective of this work is to examine the contribution made by clinical trials to strengthen the health services involved and the various components of the health system in general, namely, governance, human resources, health information, equipment, and the provision of care. By examining clinical trial site activities, skill levels, and procedures after the end of the trials, we determined the impact on routine health care of the extensive support given outside of specific trial activities and were able to observe any improvements.

## 2. Materials and Methods

**Figure 1:**
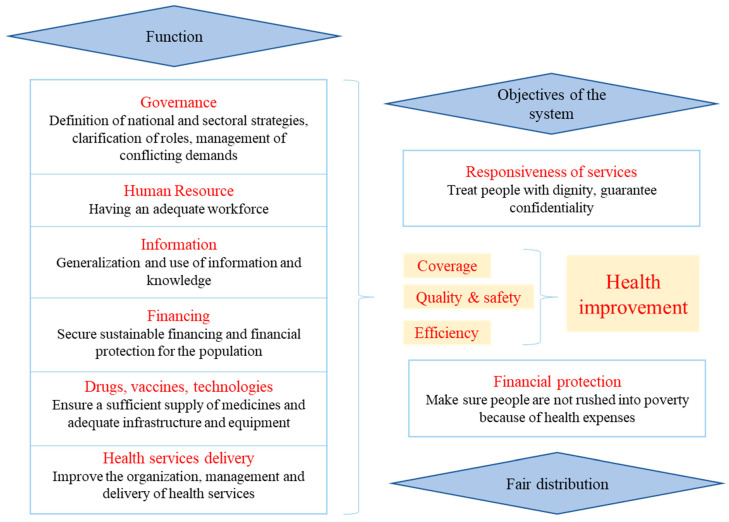
Health system functions according to WHO.

We analyzed the contribution of HAT clinical trials in terms of the six health system pillars, focusing on activities conducted during studies in the DRC. The World Health Organization (WHO) conceptual framework approaches the health system as a six-pillar package [3] in Figure 1:

Within this framework, the health system is strengthened by acting on its six pillars in order to improve health services and the health of the population in a sustainable and equitable way. A global vision is needed, and action must be taken in a balanced way to avoid compromising any successes [3].

## 3. Results 

### 3.1. Leadership and Governance

A consultation organized by WHO in 2007 found that leadership and management capacities are currently inadequate in both the private and public sectors and that few low-income countries are concerned with systematic management issues [4]. For these clinical trials, capacity was built in terms of training and further coaching of all members of the research team. The study’s national coordinating team solicited the commitment of provincial and local health authorities not to move staff who were involved in the clinical trials.

### 3.2. Human Resources

To be effective, a health system must have a critical mass of human and material resources. This resource gap is particularly keenly felt when it comes to human capital—the cornerstone of a successful health system [3]. Staff (doctors, nurses, and laboratory technicians) from 10 hospitals participated in the fexinidazole study (Bandundu, Mushie, Bagata, Vanga, Masimanimba, Dipumba, Katanda, Isangi, Dingila, and Batangafo in Central African Republic(CAR). The staff involved in the clinical trials were trained on good clinical and laboratory practice as well as on hospital waste management and universal standard precautions, in accordance with the international standards required for clinical trials. In collaboration with the HAT Platform (a clinical research and access-supporting network that brings together key regional actors involved in the control of HAT in endemic countries, notably ministries of health, national control programs, regulatory agencies, academia, clinicians, WHO, and NGOs), clinical or operational trials have also contributed to capacity building in endemic countries through the training of researchers, monitors, and practitioners on good clinical practice, laboratory diagnosis, pharmacovigilance, and patient examination techniques, including specific testing techniques. Since the creation of the HAT platform in 2005, more than 400 people have been trained in Africa in 22 training sessions with the support of universities and other African and foreign research institutions [5]. Table 1 shows the different training sessions organized in order to support clinical trials within the health service, both for the fexinidazole trials, with which this paper is primarily interested, and also for previous trials dating from 2006.

Regular supervision was carried out in the test sites, which resulted in an improvement in the quality of care. During these supervisions, each provider was assessed using an evaluation grid. If the nurse under assessment failed 20 percent or more of the tasks in question, they then received additional training and practical support.

### 3.3. Organization of Services

Even when the necessary input and enough financial support are provided, failures in service provision occur, which are often due to the dysfunctional organization of the health system [6]. Both the overall configuration of health systems and the structure of service delivery need to be addressed. For the HAT trials, the research team established a work structure with tasks for each professional category from the local investigator, nurses, and laboratory technicians to the on-duty cleaner. Training, supervision, and monitoring visits to the field research teams made it possible to improve the organization of the health service not only within the research teams but also in other departments of the hospitals involved. External monitoring and supervision were performed by a team from the Swiss Tropical and Public Health Institute.

### 3.4. Organization of Health Information

Health information is vital for decision-making and the monitoring and evaluation of the performance of the health system. In the HAT trials, research teams were reminded of the importance of documentation at all levels (investigators, nurses, and laboratory technicians) with the key maxim: "If it isn’t written down, it doesn’t exist”. The archiving of study subjects’ case report files was improved by means of the visits conducted by the clinical monitors and the information, education, and communication systems facilitated by the trial clinical coordination team based in Kinshasa.

### 3.5. Material Resources

To be efficient, a health system must have a critical mass of material resources [3]. The trials were planned at existing treatment centers in the most endemic areas. Given the limitations of the health system in DRC, each center was assessed to determine its specific material needs so that adequate conditions for conducting a clinical trial could be guaranteed. Material needs were met by providing food, guaranteeing accommodation, and facilitating personal hygiene for staff and patients. Adequate office space for the research team and trial documentation was provided, as was waste management, energy, and communication equipment. Laboratories were refurbished (Table 2). The overall cost of preparing a single hospital for the clinical trial varied between USD 20,000 and 100,000, depending on the different needs of the sites; some had recently been rehabilitated, and others were located in cities with electricity and water supplies. 

Medical equipment such as defibrillators, tools such as the PiccoloXpress analyzer (an easy-to-use fully automated system for biochemistry blood testing), and sophisticated laboratories was supplied. Internet access allowed for the transmission of case report forms, which are essential for monitoring safety parameters, including distance reading of ECG. This has helped to improve the overall care environment in the health structures involved [7]. These improvements in infrastructure supported or sustained other activities after the end of the studies. The introduction of a microscope with a video-camera in all study structures has enabled the storing of information about HAT cases, given that the best way to identify the mobile parasites is usually in fresh samples that cannot be physically stored. This microscope, together with the archiving computer program that allows for quick transmission of the images taken, plays a very important role in quality assessment and for the training of new staff. After the end of the HAT trials, this equipment may be used for the detection of other endemic diseases, such as malaria.

### 3.6. Funding

Countries in the African region are facing enormous difficulties in terms of health financing. Financial resources are inadequate, poorly managed, not strategically allocated, and not well-coordinated with what is provided by external sources [8]. The investigators were trained and supported in the efficient management of the funds placed at their disposal. Most of the HAT trial sites used the training to improve financial management and staffing of their respective hospitals. Funding was important for these trials, not only for improving the general clinical trial site environment, but also in terms of supervision visits and monitoring of field research teams who require significant logistical resources (boat, motorcycle, vehicles), and for the referral of patients who often live far from the trial sites. When providing new equipment, caution was taken to provide goods with low maintenance costs. However, it was not feasible to use the biochemistry analyzer after the trial, due to the high individual cost of the reagent discs. Instead, it was recovered after the end of the trials for use where there was external support, including in other clinical trials (Table 3).

## 4. Discussion

Most African countries face challenges related to material and human resources, health information, health financing, and the organization of service delivery. A sector-wide approach (SWAp), reported in six countries (Ghana, Malawi, Mozambique, Tanzania, Uganda, and Zambia), has been taken as an example of how to face the challenge of strengthening health systems and health system reform [3].

For the future, there are political opportunities, both in countries and regional bodies, as well as opportunities for international and technical partnerships [3]. There are opportunities for clinical and operational research within health services of low- and middle-income countries, enabling them to capitalize as much as possible on decisive progress in strengthening health systems [3].

We here describe a case study on the impact of clinical trials on the strengthening of health services, using the WHO pillar framework. These trials on fexinidazole for HAT were conducted by the Drugs for Neglected Diseases initiative (DND*i*) between 2012 and 2017 in collaboration with the Ministry of Health of DRC and Central African Republic, through their Sleeping Sickness National Control Programs. These clinical trials took place in 10 hospitals in 5 provinces of the DRC (Kwilu, Maï-Ndombe, Kasaï Oriental, Tshopo, and Bas-Uélé) and one site in Batangafo, Central African Republic. The results of the main study have been published in The Lancet [9]. 

In addition to the stated aim of developing trypanocidal drugs, several pillars of the health system were strengthened before and during clinical trials, improving the provision of services to populations living in rural areas or HAT endemic villages where the health system is deficient. The training enabled the investigating physicians, laboratory technicians, and nurses to systematically examine the health problems of the target population, to take better care of the patients included in the study, including those with problems other than sleeping sickness. Provision of additional technical equipment helped with the care of other patients not included in clinical trials, and the use of new diagnostic tools improved levels of trust for medical staff amongst patients.

Watson Tawaba, a nurse in a clinical trial site in the DRC said: "With the clinical trial on fexinidazole, everything has changed. Not only does our hospital no longer look like a farm, but the community benefits from a modern facility, and our work is easier" [10].

In the former Bandundu province in DRC, hospital staff involved in HAT clinical trials have increased the capacity of staff at other rural hospitals not involved in clinical trials in terms of patient record-keeping and the execution of nursing techniques such as venous catheterization, as reported by the North Bandundu Trypanosomiasis Coordination (2009–2012, unpublished report). Prior to the HAT trials, trypanosomiasis unit nurses in one of the clinical trial sites would request support from pediatric nurses for the venous catheterization of patients. After training in the introduction of venous catheters, the research team nurses began to assist their pediatric colleagues in the placement of venous catheters in children and in the handling of resuscitation devices or oxygen concentrators.

The research teams have become an example within their hospitals because they have been well-trained. For remaining staff who were not involved in the study, there is still a long way to go before the improved diagnostic tools, standard precautions, and evidence-based medical care, including careful attention to the patient’s clinical evolution, are strictly implemented in the hospitals.

The goal of the training was for clinical trial teams to be able to include, treat, and follow patients in accordance with protocol (goal achievement). The investigators had to adapt to the rules and principles that clinical trials impose (adaptation to the research environment) and maintain the functionality of the health facilities (organizational culture).

**Figure 2:**
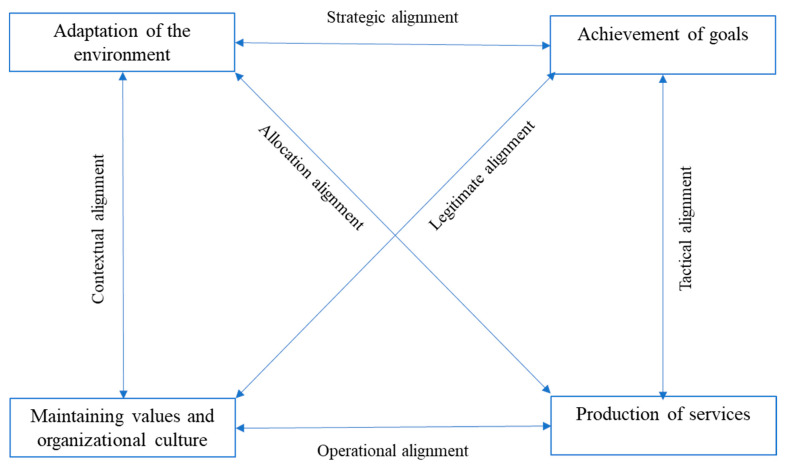
Four functional systems of health care organizations and their six interchange subsystems [11].

Using the impact analysis framework in Figure 2, we found that the HAT clinical study was successful because it considered the dynamic balance of all functions [11].

In these clinical trials, patients with HAT were recruited according to inclusion and exclusion criteria after obtaining informed consent. These patients were diagnosed either by hospitals involved in clinical trials or by mobile teams who actively screen at-risk populations in endemic villages to identify patients who were subsequently referred to clinical trial sites. In 2016–2017, DRC’s Programme National de Lutte contre la Trypanosomiase Humaine Africaine (PNLTHA) mobile screening teams, supported by DND*i*, which were active around trypanocidal drug trial sites, examined one-third of the 2 million people examined annually nationwide. A strengthening of the current network of passive screening set up by the PNLTHA around clinical trials research centers is underway. This measure should not only makes it possible to identify additional patients for clinical trials but also to create a passive surveillance system for the sustainable elimination of trypanosomiasis [7].

## 5. Conclusions

DND*i*’s experience of conducting clinical trials in collaboration with the national sleeping sickness program in the DRC through the support of the HAT platform has shown that it is possible to create an enabling environment in an endemic country for the conduct of quality clinical trials. Conducting clinical trials in endemic areas has not only reinforced and supported the aim of strengthening high-level clinical research, but has also brought lasting benefits to researchers, staff, and hospitals, as well as to broader health systems. This has a positive knock-on effect on patients outside of the clinical trials and their communities because clinical trial physicians and nurses or lab technicians have shared their new knowledge and experience in governance, GCP, and waste management with staff in their own and other hospitals [7].

When planning to set up this clinical trial, the epidemiology of the target disease was assessed, and this information was used to direct the process of site selection. The trial was conducted in existing health structures that were already treating patients, which facilitated trial execution. Adequate material resources and technologies were needed to ensure that the trial could be conducted up to international research standards and fulfill the requirements of good clinical practice. The sustainability of the improvements after the end of the trial depends mainly on infrastructure improvements, added knowledge, and improved staff practices. The sustainability of equipment depends on the ability of local health services to fund maintenance and consumables; it was not feasible to maintain the biochemistry machine PiccoloXpress®, but other simple laboratory techniques that were introduced will certainly remain. Standard hygiene precautions and waste management require continuous supervisory input, and sustainability will depend on the motivation of the staff and the leadership at each structure.

Clinical research in most of sub-Saharan Africa remains very dependent on international input and external technical support, especially in the HAT-endemic countries of central Africa. Although efforts are being made to build capacity and infrastructure, weak points remain in terms of trial design and documentation, as well as data management and analysis. Financing is a critical issue, as African investigators find it difficult to directly access international research funding sources and rely on international partnerships.

## Figures and Tables

**Table 1:** The number of training sessions and people trained as part of HAT clinical trials since 2006 [5].

Trainings Conducted (Number of People Trained)	Venue and Year
Training in ethical review of research (142)	Kinshasa 2007
	Khartoum 2007
	Kampala 2007
	Luanda 2008
	Juba 2009
	Bangui 2010
Training of physicians in good clinical practice (GCP) (96)	Nairobi 2006, Kinshasa 2011 and 2012, Juba 2012
Training of physicians on clinical examination of the patient (25)	Kinshasa 2007
Training of clinical monitors (13)	Kampala 2008
Participation at ICAT6 and -7 (26)	Kinshasa 2014, Kampala 2017
HAT training in Dinamadji health district (30)	Dinamadji 2015
HAT clinical training in South Sudan (41)	Juba 2015
Training of Guinean physician in DRC (1)	Kinshasa 2014
Training of laboratory technicians from South Sudan in DRC (3)	Kinshasa 2016
Training of mobile team technicians on HAT diagnosis in DRC (36)	Kinshasa 2016
Waste management training at clinical trial sites in DRC (182)	Mushie, Vanga, Bagata, Masi 2016

**Table 2:** List of material needs for the improvement of clinical trial sites and how these needs were addressed.

Material Need.	Provision
Food	Provided for all HAT patients, irrespective of trial participation
Accommodation	Beds repaired or purchased; new mattresses and mosquito nets; dedicated wards repainted; floors, windows repaired
Personal hygiene	Latrines and shower blocks built; water supply arranged, including rainwater reservoirs, for the benefit of staff and patients
Office space	A nursing room, investigator’s office, lockable cupboards, and furniture were provided for the research team and trial documentation
Waste management	A closed waste disposal area with three separated pits was provided or improved; incinerators were built or rehabilitated
Laboratory space	Refurbishment through rebuilding of interiors, including working surfaces and necessary equipment
Energy	Generators and solar systems for lightning, electric equipment, and a cold chain were provided.
Communication equipment	Computers, printers, internet access, and telephone cards were provided

**Table 3:** Cost of the main material resources provided to a model hospital.

Items	Unit Cost in USD (Euro Converted at USD 1.1)
**Rehabilitation Works**	
Preparation and construction of waste areas	USD 10,150
Latrine and shower construction	USD 8730
Rainwater collection system	USD 5000
Preparation of investigators’ offices	USD 750
Lab preparation for routine exams	USD 17,500
5kva solar panels	USD 21,000
**Medical Equipment**	
Pavilion equipment with 12 beds	USD 1800
Foldable examination table	USD 164
Mechanical weight and height scale	USD 227
Life support equipment	USD 2443
Emergency bag kit	USD 811
**Laboratory Equipment**	
Microscopes with Camera	USD 3470
8-tube electric centrifuges	USD 1122
Electric Hematocrit Centrifuges	USD 1467
HemoCue Hb 301	USD 548
Eppendorf" automatic pipette	USD 242
**Cold Chain**	
Vestfrost refrigerator	USD 895
Cold chain (Freezer + specific solar panels + batteries)	USD 24,074
**Transport and Office Equipment**	
Motorbike Yamaha AG100	USD 4600
Laptop	USD 1000
Internet connection kit	USD 2760
Printer, scanner, photocopier	USD 300
